# CAR T-cell therapy response varies by extranodal disease site in large B-cell lymphoma

**DOI:** 10.1038/s41408-025-01273-1

**Published:** 2025-04-14

**Authors:** Alejandro Luna, Sean M. Devlin, Kai Rejeski, Jessica R. Flynn, Magdalena Corona, Efrat Luttwak, Alfredo Rivas-Delgado, Ivan Landego, Giulio Cassanello, Marina Gomez-Llobell, Sandeep S. Raj, Parastoo B. Dahi, Richard J. Lin, Allison Parascondola, M.Lia Palomba, Gunjan L. Shah, Michael Scordo, Ana Alarcon Tomas, Doris Leithner, Akshay Bedmutha, Heiko Schöder, Brandon S. Imber, Gilles Salles, Jae H. Park, Miguel-Angel Perales, Roni Shouval

**Affiliations:** 1 Adult Bone Marrow Transplant Service, Department of Medicine, Memorial Sloan Kettering Cancer Center, New York, NY USA; 2 Bone Marrow Transplantation Unit. Hematology Service. Hospital Universitario Ramon y Cajal, Madrid, Spain; 3 Department of Epidemiology and Biostatistics, Memorial Sloan Kettering Cancer Center, New York, NY USA; 4 Department of Medicine III, Hematology/Oncology, LMU University Hospital, LMU Munich, Munich, Germany; 5 Hematology Service, Hospital Universitario 12 de Octubre, Madrid, Spain; 6 Lymphoma Service, Memorial Sloan Kettering Cancer Center, New York, NY USA; 7 Lymphoma Unit, IRCCS San Raffaele Scientific Institute, Milan, Italy; 8 Department of Medicine, Weill Cornell Medical College, New York, NY USA; 9 Cellular Therapy Service, Memorial Sloan Kettering Cancer Center, New York, NY USA; 10 Hospital Universitario Puerta de Hierro, Madrid, Spain; 11 Department of Radiology, Memorial Sloan Kettering Cancer Center, New York, USA; 12 Department of Radiation Oncology, Memorial Sloan Kettering Cancer Center, New York, NY USA

**Keywords:** B-cell lymphoma, Risk factors

## Abstract

The role of extranodal (EN) sites as potential sanctuary regions resistant to CD19-directed chimeric antigen receptor T-cell (CAR-T) therapy in large B-cell lymphoma (LBCL) remains unclear. To investigate this, we retrospectively analyzed 283 adults treated with commercial CD19 CAR-T therapy, assessing 958 PET-CT scans across four time points: pre-apheresis, pre-lymphodepletion, best response, and relapse. EN involvement prior to CAR-T therapy was common (76%). Outcomes for patients with exclusive EN disease were similar to those with nodal (ND) disease alone; however, patients with concomitant EN and ND disease (EN + ND) had lower complete response rates and shorter progression-free survival. Site-specific outcomes varied: lungs/pleura/pericardium and gastrointestinal/peritoneum involvement had the lowest local response rates (48% and 51%, respectively). Notably, the risk of same-site relapse was highest in the lungs/pleura/pericardium (hazard ratio [HR] 7.8) and gastrointestinal/peritoneum (HR 5.97). Among patients relapsing after CAR-T, two-year overall survival rates from time of relapse were significantly lower in those with EN relapse (23% for exclusive EN; 25% for EN + ND) compared to exclusive ND relapse (64%; *p* = 0.008). These findings underscore the high prevalence of EN disease in CAR-T recipients and its site-specific impact on outcomes, highlighting the need for organ-targeted strategies to enhance treatment efficacy.

**Figure:**
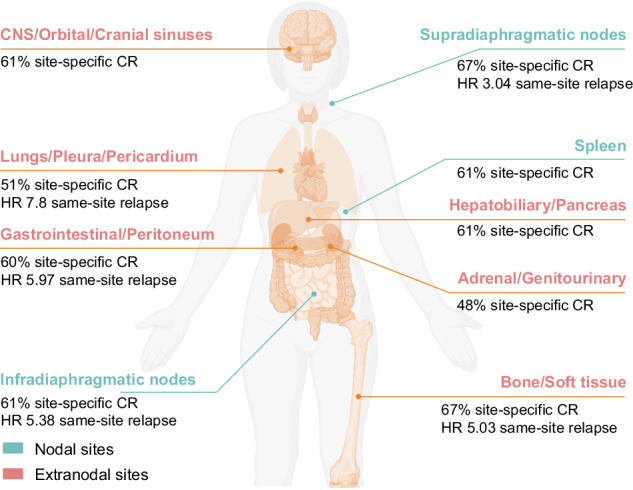
Differential site-specific response and relapse/progression risk according to pre-CAR-T therapy anatomical site involvement in Large B-cell Lymphoma. Risk of site-specific relapse or progression was not evaluable for CNS/orbital/cranial sinuses, adrenal/genitourinary, hepatobiliary/pancreas, and spleen due to insufficient number of events.

Differential site-specific response and relapse/progression risk according to pre-CAR-T therapy anatomical site involvement in Large B-cell Lymphoma. Risk of site-specific relapse or progression was not evaluable for CNS/orbital/cranial sinuses, adrenal/genitourinary, hepatobiliary/pancreas, and spleen due to insufficient number of events.

## Introduction

Chimeric antigen receptor T-cell (CAR-T) therapy has transformed the care of relapsed or refractory large B-cell lymphoma (LBCL) [1–3]. While CAR-T therapy achieves significant remission rates, up to 60% of patients experience relapse or refractory disease, highlighting ongoing challenges [4–6]. These outcomes reflect a complex interplay among host factors, CAR-T product attributes, and disease-specific characteristics [7–10]. Among these, tumor-related features—such as disease burden and lymphoma-specific genomic alterations—have emerged as important determinants of response to CAR-T therapy [1–3, 11–13]. However, variability in disease control among CAR-T recipients remains poorly understood, necessitating further investigation.

The inherent heterogeneity of LBCL is further compounded by variations in disease involvement across anatomical sites [14–16]. Differences in organ tropism, driven by genetic and phenotypic heterogeneity, may significantly influence treatment response [17–21]. Extranodal involvement has been widely recognized as an adverse prognostic factor in the first-line treatment of LBCL, with certain regions—such as the central nervous system (CNS), respiratory tract, liver, and pancreas—associated with particularly poor outcomes [15–17]. However, data on the influence of anatomical disease distribution in patients receiving CAR-T therapy are limited, with most studies focusing primarily on CNS involvement [22–25]. The broader role of anatomical sites in shaping treatment response remains unclear and warrants further investigation.

In this retrospective observational study, we hypothesized that specific anatomical sites may act as protective sanctuaries from CAR-T therapy. To test this hypothesis, we analyzed LBCL site involvement patterns before and after CAR-T therapy and evaluated their implications on key clinical outcomes.

## Methods

### Patient cohort and design

We conducted a retrospective analysis of 283 adult patients (age ≥18 years) with relapsed or refractory LBCL who received CAR-T therapy at Memorial Sloan Kettering Cancer Center (MSK) from January 2018 to September 2023. Patients with primary CNS involvement and patients with no evidence of disease at the time of pre-apheresis assessment were excluded from the study. CAR-T products were administered as standard of care (axicabtagene ciloleucel [axi-cel], tisagenlecleucel [tisa-cel], and lisocabtagene maraleucel [liso-cel]), except for liso-cel infusions, some of which were performed as part of a clinical trial (NCT02631044) [3].

Patient data were recorded and entered into a REDCap database [26].

### Treatment response and outcome

Treatment response was assessed according to Lugano classification [27]. The best overall response was defined as the most favorable response, achieved at any time from CAR-T cell infusion up to day 365. Overall survival (OS) and progression-free survival (PFS) were measured from the time of CAR-T cell infusion; corresponding events were death for OS and death, relapse, or disease progression for PFS. Patients without events were censored at their date of last follow-up. Relapse and progression were estimated using cumulative incidence functions with death as a competing risk. Site-specific relapse or progression was also analyzed using cumulative incidence functions; however, for these outcomes, competing risks were death along with relapse or progression in a site other than the one under investigation. Site-specific complete response to CAR-T therapy was defined as the absence of disease at previously involved sites during the best response assessment. Cytokine release syndrome (CRS) and immune effector cell-associated neurotoxicity syndrome (ICANS) were defined according to the American Society of Transplantation and Cellular Therapy criteria [28].

### Evaluation of site-specific disease involvement

**Fig. 1:**
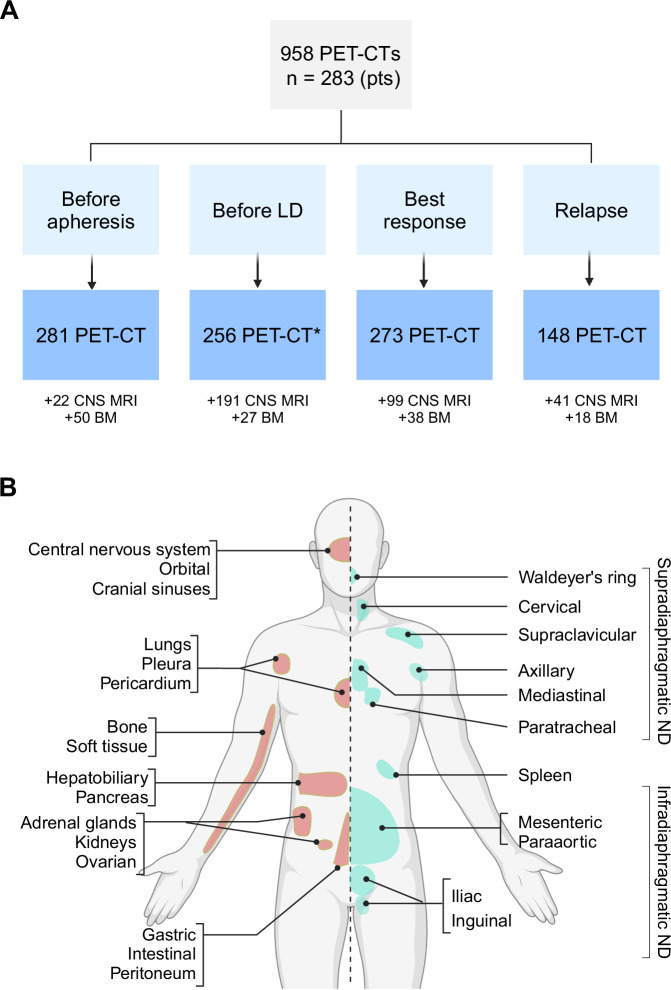
Disease evaluation and definitions. **A** Disease involvement was assessed at four time points using PET-CT and additional modalities, as outlined in the flow diagram. **B** Definitions for clustering of nodal and extranodal disease sites and localization by supradiaphragmatic and infradiaphragmatic nodes for ND disease. * Of 256 patients with available PET-CTs following apheresis, 231 were evaluated after bridging therapy. LBCL Large B-cell Lymphoma, LD lymphodepletion, PET-CT positron emission tomography/computed tomography, CNS central nervous system, MRI magnetic resonance imaging, BM bone marrow.

Disease involvement was systematically evaluated at four key time points: prior to apheresis, prior to lymphodepletion (LD), at best response, and at relapse or progression (Fig. 1A). A total of 958 18F-fluorodeoxyglucose positron emission tomography/computed tomography (PET-CT) scans were analyzed, including 281 pre-apheresis, 256 pre-LD, 273 at best response, and 148 at relapse or progression. Data extracted from PET-CT reports included the number and location of lesions, classification of sites as nodal (ND) or extranodal (EN), and disease staging. ND sites were defined according to the Revised European-American Lymphoma (REAL) classification, encompassing lymph nodes, spleen, thymus, and Waldeyer’s ring [15].

CNS involvement was evaluated using magnetic resonance imaging (MRI), with 351 scans reviewed, including 213 conducted prior to CAR-T therapy. CNS imaging was performed based on a clinical indication or suspicion, or for screening purposes. Response in patients with CNS involvement was assessed based on radiographic evaluation by a radiologist using PET-CT and MRI.

Disease involvement was categorized into three groups before CAR-T therapy: exclusively ND disease, exclusively EN disease, and concomitant EN and ND disease. Bone marrow involvement was not included in these categories since it was assessed in a small fraction of patients (*n* = 43). Bone disease was determined exclusively based on FDG uptake using standard radiographic criteria and considered as EN disease.

Anatomical site involvement was clustered as follows (Fig. 1B): CNS with orbital and cranial sinuses, lungs with pleura and pericardium, gastrointestinal with peritoneum, hepatobiliary with pancreas, adrenal glands with kidneys and ovarian, and bone with soft tissue. Additionally, nodal involvement was classified into supradiaphragmatic regions (cervical, supraclavicular, axillary, mediastinal, and hilar/paratracheal lymph nodes) and infradiaphragmatic regions (mesenteric/paraaortic, iliac, and inguinal lymph nodes). Spleen involvement was considered separately from the other nodal regions.

Metabolic tumor volume (MTV) and standardized uptake value (SUV) were calculated as previously described using PET-CT scans [29]. Same-site relapse or progression was defined as the reappearance or worsening of metabolic activity in the same anatomical location as prior tumors, based on radiologists assessment of increased SUV or other indicators of disease activity; death was considered a competing event.

### Sequencing

Integrated Molecular Profiling and Analysis for Clinical Tumors (IMPACT) sequencing was used to identify somatic alterations in tumor samples. DNA was extracted from formalin-fixed, paraffin-embedded tumor tissues and matched non-involved samples, which were available for 118 patients prior to CAR-T therapy. Barcoded libraries were prepared, and sequencing targeted all exons and selected introns of approximately 400 cancer-related genes using a custom gene panel. Sequencing data were processed using an established bioinformatics pipeline to detect somatic mutations, gene fusions, and copy number alterations [30]. Genomic data were curated and uploaded to the cBioPortal platform for integrative analysis [31].

### Statistical analysis

Continuous variables were summarized using the median and interquartile range, while categorical data were presented as frequencies and percentages.Tests of association were based on a Fisher’s exact test, Chi-square test, or a Wilcoxon rank-sum test, as appropriate. Cox proportional hazards models were used to analyze associations with OS and PFS, while logistic regression models were applied to grade 2–4 CRS, grade 2–4 ICANS, response to bridging therapy, and complete response (CR) to CAR-T therapy. Cause-specific Cox regression models estimated univariable and multivariable models of overall relapse and site-specific relapse. *P*-values below 0.05, including adjusted *p*-values, were considered statistically significant. All analyses were conducted using R statistical software (version 4.3, R Foundation for Statistical Computing).

### Ethics approval and consent to participate

This retrospective study was approved by the Memorial Sloan Kettering Cancer Center Institutional Review Board (IRB), approval number 19–373. All methods were performed in accordance with relevant guidelines and regulations. The requirement for informed consent was waived by the IRB, as this retrospective study involved minimal risk to participants and did not adversely affect their rights or welfare.

## Results

### Population characteristics

We included 283 patients with a median age of 66 years (IQR: 56–72) who were treated with commercial CAR-T cell therapy (58% axi-cel, 24% tisa-cel, and 19% liso-cel) (Table 1). Diffuse large B-cell lymphoma Not Otherwise Specified was the most common diagnosis (77%), followed by high-grade B-cell lymphoma (16%) and other LBCL subtypes (7%). Transformed LBCL was reported in 36% of patients. At the last assessment before CAR-T infusion, 40% of patients had elevated LDH, and the median MTV was 23 mL (IQR: 1–141). Bridging therapy was administered to 82% of patients. CAR-T therapy was used as a third-line or later treatment in 80% of patients.

**Table 1:** Population characteristics by EN Disease involvement at last assessment before CAR-T therapy.

Characteristic	*N*	Overall *N* = 283	Exclusive ND *N* = 64	Exclusive EN *N* = 66	Concomitant EN and ND *N* = 133	No evidence of disease (CR) *N* = 20	p-value for comparison of ND vs. EN vs concomitant EN + ND*
Median Age [years]	283	66 (56, 72)	64 (55, 70)	66 (55, 70)	67 (56, 74)	69 (57, 77)	0.2
Sex	283						0.8
Male		184 (65%)	44 (69%)	42 (64%)	90 (68%)	8 (40%)	
Female		99 (35%)	20 (31%)	24 (36%)	43 (32%)	12 (60%)	
Karnofsky Performance Status	283						0.009
>=90		80 (28%)	27 (42%)	19 (29%)	28 (21%)	6 (30%)	
<90		203 (72%)	37 (58%)	47 (71%)	105 (79%)	14 (70%)	
LBCL subtypes	283						0.12
DLBCL NOS		219 (77%)	53 (83%)	52 (79%)	98 (74%)	16 (80%)	
High-grade B-cell lymphoma		44 (16%)	4 (6.3%)	11 (17%)	25 (19%)	4 (20%)	
Other LBCL^†^		20 (7.1%)	7 (11%)	3 (4.5%)	10 (7.5%)	0 (0%)	
Transformed LBCL	279						0.12
De-novo LBCL		178 (64%)	45 (70%)	45 (69%)	75 (57%)	13 (68%)	
Transformed LBCL		101 (36%)	19 (30%)	20 (31%)	56 (43%)	6 (32%)	
Unknown		4	0	1	2	1	
Cell of origin	273						0.6
Non-GCB		132 (48%)	26 (43%)	34 (52%)	60 (47%)	12 (67%)	
GCB		141 (52%)	34 (57%)	32 (48%)	69 (53%)	6 (33%)	
Unknown		10	4	0	4	2	
Double hit status	263						0.2
Not Double/Triple Hit		223 (85%)	56 (92%)	53 (87%)	100 (81%)	14 (78%)	
Double/Triple Hit		40 (15%)	5 (8.2%)	8 (13%)	23 (19%)	4 (22%)	
Unknown		20	3	5	10	2	
MYC rearrangement	263	64 (24%)	6 (9.8%)	18 (30%)	34 (28%)	6 (33%)	0.009
Unknown		20	3	5	10	2	
Stage at apheresis	281						<0.001
<=II		81 (29%)	33 (52%)	18 (28%)	23 (17%)	7 (35%)	
III-IV		200 (71%)	31 (48%)	47 (72%)	109 (83%)	13 (65%)	
Unknown		2	0	1	1	0	
Pre-lymphodepletion LDH	283						0.001
normal		169 (60%)	46 (72%)	44 (67%)	63 (47%)	16 (80%)	
elevated		114 (40%)	18 (28%)	22 (33%)	70 (53%)	4 (20%)	
Metabolic Tumor volume (last assessment) [cm^3^]	244	23 (1, 142)	15 (1, 53)	3 (0, 62)	91 (16, 369)	0 (0, 0)	<0.001
Unknown		39	10	10	15	4	
Pre-apheresis treatment lines	280						0.088
<=3 lines		192 (69%)	49 (78%)	38 (59%)	90 (68%)	15 (75%)	
> 3 lines		88 (31%)	14 (22%)	26 (41%)	43 (32%)	5 (25%)	
Unknown		3	1	2	0	0	
Previous autologous transplantation	283	55 (19%)	15 (23%)	16 (24%)	20 (15%)	4 (20%)	0.2
Pre-apheresis primary refractory disease	283	114 (40%)	29 (45%)	18 (27%)	60 (45%)	7 (35%)	0.038
Bridging^#^	283	231 (82%)	48 (75%)	52 (79%)	113 (85%)	18 (90%)	0.2
Bridging type^#^	283						0.006
No bridging		52 (18%)	16 (25%)	14 (21%)	20 (15%)	2 (10%)	
Systemic bridging / steroids		181 (64%)	30 (47%)	38 (58%)	97 (73%)	16 (80%)	
Non-systemic bridging		50 (18%)	18 (28%)	14 (21%)	16 (12%)	2 (10%)	
Pre-infusion disease status	283						<0.001
CR		21 (7.4%)	2 (3.1%)	1 (1.5%)	0 (0%)	18 (90%)	
PR		99 (35%)	26 (41%)	37 (56%)	34 (26%)	2 (10%)	
SD/PD		163 (58%)	36 (56%)	28 (42%)	99 (74%)	0 (0%)	
CAR-T Product	283						>0.9
Axicabtagene ciloleucel		163 (58%)	39 (61%)	38 (58%)	77 (58%)	9 (45%)	
Tisagenlecleucel		67 (24%)	13 (20%)	16 (24%)	32 (24%)	6 (30%)	
Lisocabtagene maraleucel		53 (19%)	12 (19%)	12 (18%)	24 (18%)	5 (25%)	
CRP at infusion [mg/dl]	280	1.2 (0.5, 3.5)	0.9 (0.5, 2.7)	0.7 (0.5, 1.8)	2.1 (0.8, 7.1)	0.5 (0.4, 0.9)	<0.001
Unknown		3	1	1	1	0	
Ferritin at infusion [ng/ml]	280	270 (115, 695)	180 (109, 460)	205 (80, 530)	453 (143, 1,077)	246 (81, 464)	<0.001
Unknown		3	0	1	2	0	
IL6 at infusion [pg/ml]	220	9 (5, 21)	8 (5, 13)	6 (3, 12)	13 (5, 33)	5 (3, 8)	<0.001
Unknown		63	12	20	27	4	
Median (Q1, Q3); n (%)							

^*^Kruskal-Wallis rank sum test; Pearson’s Chi-squared test; Fisher’s exact test; *P*-value reflects the comparison between exclusive ND, exclusive EN, and concomitant EN and ND disease.

#Bridging therapy was defined as any systemic or localized treatment administered between leukapheresis and lymphodepleting chemotherapy with the intent to control disease before CAR-T infusion. Systemic bridging included chemotherapy, targeted agents, corticosteroids, or immunotherapy, while localized therapy included radiotherapy or intrathecal chemotherapy. The decision to administer bridging therapy and the choice of regimen were at the discretion of the treating physician, guided by disease burden, progression risk, and institutional practice. Further description of bridging therapies is provided in Table S4.

†Other LBCL categories include: primary mediastinal B-cell lymphoma (*n* = 10), EBV-positive diffuse large B-cell lymphoma (*n* = 6), T-cell/histiocyte-rich B-cell lymphoma (*n* = 3), intravascular large B-cell lymphoma (*n* = 1).

*EN* extranodal, *ND* nodal, *LBCL* large B-cell lymphoma, *DLBCL* diffuse large B-cell lymphoma, *GCB* germinal center B-cell, *LDH* lactate dehydrogenase, *CR* complete response, *PR* partial response, *SD* stable disease, *PD* progressive disease.

CRS occurred in 42% of patients, with grade ≥2 CRS in 29%. ICANS was observed in 29%, with grade ≥2 ICANS in 20%. The overall response rate (ORR) to CAR-T therapy was 80%, with a CR rate of 64%. With a median follow-up of 26 months (IQR: 13–49), 2-year PFS and OS were 36% (95% CI: 30–43%) and 53% (47–60%), respectively. At 2 years, the cumulative incidence of relapse or progression was 58% (51–64%), and non-relapse mortality was 7.4% (4.5–11%).

### Extranodal disease status and response to bridging

At apheresis, 79 patients (25%) had ND disease only, 67 patients (24%) had exclusive EN disease, and 137 patients (51%) had concomitant EN and ND disease. Among the 231 patients who received bridging therapy, 64% underwent systemic treatment (with or without localized treatment, such as radiotherapy or intrathecal chemotherapy), while 18% received exclusively localized monotherapy.

To minimize bias related to the selection of bridging modalities, analyses were restricted to patients receiving systemic bridging therapy (Fig. S1). Among patients with evaluable post-bridging disease assessments (*n* = 175), the ORR to systemic bridging therapy was 47% (9% CR and 38% partial response). For patients with exclusive ND disease involvement pre-apheresis, the ORR to bridging therapy was 46% (CR 14%). ORR for patients with exclusive EN disease was 54% (CR 9%), and for those with concomitant EN and ND disease was 45% (CR 7%). In univariable logistic regression, neither exclusive EN disease nor concomitant EN and ND disease was significantly associated with overall response to bridging therapy (OR 0.73 [95% CI: 0.29–1.79] and OR 1.04 [0.25–2.15], respectively).

### Patients with concomitant extranodal and nodal disease have unfavorable outcomes

At the last disease assessment before CAR-T infusion, 263 out of 283 (93%) had evidence of disease; 64 out of 263 patients (24%) had exclusive ND disease, while the remaining 199 patients (76%) exhibited some EN involvement. EN disease typically co-occurred with ND disease (133/199, 67%), with a smaller subset presenting with exclusive EN disease (66/199, 33%).

Patients with concomitant EN and ND involvement exhibited adverse prognostic factors such as lower Karnofsky performance status, elevated LDH levels pre-lymphodepletion, and higher MTV (Table 1). The most frequently observed mutations (Fig. S2, Table S1) in the patients with any EN disease involvement were *KMT2D* (40%), *TP53* (38%), and *CDKN2A* (24%).

Rates of grade ≥ 2 CRS occurred in 50% of exclusive EN cases, 47% of concomitant EN and ND cases, and 31% of exclusive ND cases (*p* = 0.062). Grade ≥ 2 ICANS was more frequent in patients with EN involvement, appearing in 27% of both exclusive EN and combined EN and ND groups, compared to 6% in the exclusive ND group (*p* = 0.002). For patients with CNS/orbital/cranial sinus involvement at the last assessment, 38% experienced ICANS, versus 20% among those without CNS involvement (*p* = 0.093).

CR rates to CAR-T therapy were 68% for patients with exclusive ND disease, 70% for those with exclusive EN disease, and 59% for those with concomitant EN and ND disease. In multivariable logistic regression, adjusted for age, transformed lymphoma, KPS, pre-LD LDH, bridging therapy, and CAR-T product, patients with concomitant EN and ND disease had a significantly higher risk of not attaining CR compared to those with exclusive ND disease (OR 2.13 [1.10–4.23], *p* = 0.028). In contrast, patients with exclusive EN disease did not exhibit a significant difference (OR 1.17 [0.54–2.53], *p* = 0.7).

**Fig. 2:**
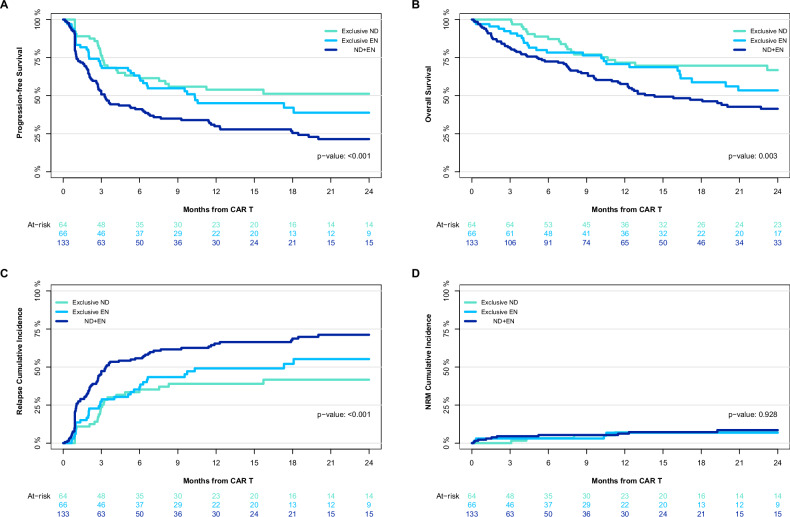
Patients with concomitant EN and ND disease at last assessment before CAR-T therapy have unfavorable outcomes. Progression-free survival (**A**), and overall survival (**B**), relapse (**C**), and non-relapse mortality (NRM) **(D**) by nodal or extranodal disease status at last assessment before CAR-T therapy.

PFS at 2 years post-CAR-T infusion was highest in the exclusive ND group (51% [95% CI: 40–60%]), followed by 39% (27–55%) in the exclusive EN group, and 21% (15–31%) in the concomitant EN and ND group (*p* < 0.001) (Fig. 2A). In multivariable Cox regression adjusted for the same variables as above, concomitant EN and ND disease was associated with significantly lower PFS (HR 1.72 [1.12–2.65], *p* = 0.014) compared to exclusive ND. In contrast, there was no evidence that the PFS risk was increased with exclusive EN disease (HR 1.23 [0.76–1.98], *p* = 0.4).

Two-year OS was also highest in the exclusive ND group (67% [55%-81%]), followed by 53% (41–70%) for exclusive EN, and 41% (33–52%) for concomitant EN and ND (*p* = 0.003) (Fig. 2B). In multivariable Cox regression, neither concomitant EN and ND disease (HR 1.52 [0.91–2.53], *p* = 0.11) nor exclusive EN disease (HR 1.30 [0.73-2.31], *p* = 0.4) were associated with a significantly shorter OS compared to exclusive ND.

Cumulative incidence of 2-year relapse or progression were 42% (29–54%) for ND versus 55% (40–68%) for exclusive EN versus 71% (62–79%) for concomitant EN-ND (*p* < 0.001, Fig. 2C). In multivariable cause-specific Cox regression, concomitant EN and ND disease had a higher risk of relapse or progression (HR 1.98 [1.23-3.12], *p* = 0.003) compared to exclusive ND disease. In contrast, the risk was not increased with exclusive EN disease (HR 1.32 [0.79–2.21]) compared to ND disease. The cumulative incidence of non-relapse mortality at 2 years post-infusion was 7.8% (3.2–15%) for exclusive ND, 9.1% (4.5–16%) for concomitant EN and ND, and 3.5% (0.63–11%) for exclusive EN (Fig. 2D)

In a sensitivity analysis replacing LDH with MTV in multivariable models, concomitant EN + ND remained associated with a higher risk of relapse or progression compared to exclusive ND disease (HR 1.72 [1.05–2.81], *p* = 0.003). Exclusive EN was associated with shorter OS (HR 1.92 [1.03–3.56], *p* = 0.04) and a numerically higher, but not statistically significant, risk of not achieving CR (OR 1.82 [0.75–4.52], *p* = 0.2). Concomitant EN + ND showed a trend toward worse PFS but did not reach statistical significance (HR 1.40 [0.88–2.22], *p* = 0.2).

### CAR-T outcome is related to the number of extranodal sites

Next, we investigated the relationship between the number of EN sites at the last assessment before CAR-T and CAR-T outcomes, irrespective of nodal involvement. In the exclusive EN group, 49% had a single EN site, 15% had two sites, and 36% had three or more. In the concomitant EN + ND group, the corresponding rates were 26%, 20%, and 53% (*p* = 0.01).

Patients with one, two, and three or more EN disease sites before CAR-T therapy had CR rates of 62%, 72%, and 43%, respectively. Involvement of three or more sites was strongly related to shorter OS and PFS and higher relapse, while these were comparable between patients with one or two sites of disease (Tables S2 and S3).

### Differential response rates across disease sites

**Fig. 3:**
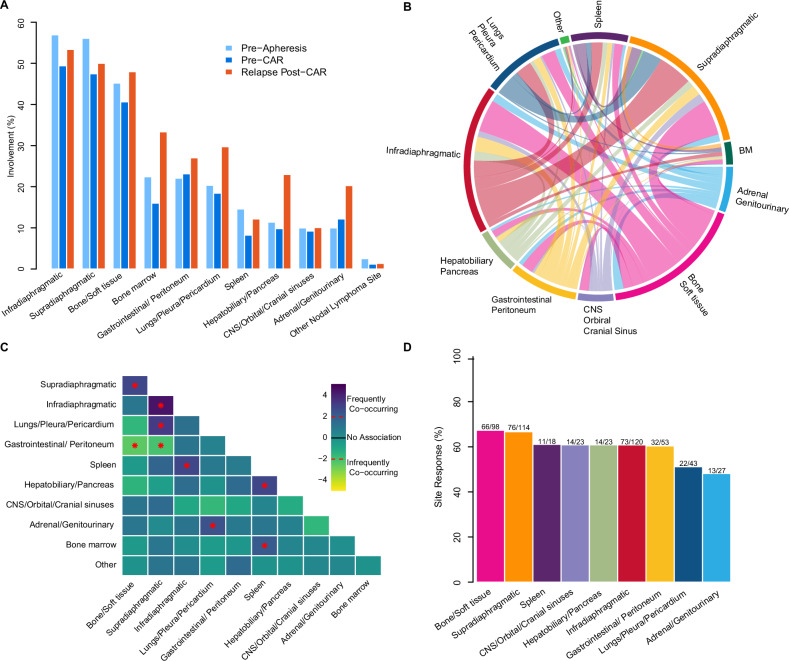
Anatomical site distribution, correlation patterns, and response. **A** Distribution of disease involvement sites at pre-apheresis, last assessment, and relapse/progression. **B** Circus diagram showing the relative frequency and pairwise co-occurrence of anatomical sites involved with lymphoma before apheresis. Arc length represents the frequency of site involvement, while ribbon width indicates the percentage of patients with co-occurrence at a second site. **C** Pairwise association analysis between anatomical sites involved before apheresis using logistic regression. The color represents the z-statistics from the model. Values above 1.96 correspond to frequently co-occurring pairs of sites, and values below -1.96 correspond to infrequently co-occurring pairs. The red * represents statistical significance (*p* < 0.05) using the z-statistic. **D** Site-specific response rates to CAR-T therapy. Response is assessed relative to disease involvement at last disease assessment before CAR-T therapy. BM bone marrow, GI gastrointestinal, CNS central nervous system.

Infradiaphragmatic and supradiaphragmatic regions were the most frequently involved sites, with ≥45% of patients affected both before apheresis and at the last disease assessment prior to CAR-T therapy (Fig. 3A). Commonly involved EN sites at both time points included bone/soft tissue (40–45%), gastrointestinal/peritoneum (22–23%), lungs/pleura/pericardium (18–20%), and hepatobiliary/pancreas (10–11%). CNS/orbital/cranial sinuses involvement was less frequent, observed in 8–9% of patients.

At pre-apheresis disease assessment, strong associations between specific sites were identified: supradiaphragmatic nodes were correlated with bone/soft tissue and lungs/pleura/pericardium; infradiaphragmatic nodes were associated with the spleen; lungs/pleura/pericardium correlated with adrenal/genitourinary involvement; and the spleen correlated with hepatobiliary/pancreas involvement (Fig. 3B, C).

Site-specific CR rates to CAR-T therapy varied significantly. The highest responses were observed in bone/soft tissue (67%), supradiaphragmatic nodes (66%), and spleen (61%). In contrast, lower CR rates were noted for adrenal/genitourinary sites (48%), lungs/pleura/pericardium (51%), and CNS/orbital/cranial sinuses (58%) (Fig. 3D). Notably, in a subanalysis considering CNS involvement, without combining it with orbital or cranial sinuses involvement, site-specific CR rates were 50% among 16 evaluable patients.

Next, we evaluated whether site involvement at the last assessment before CAR-T therapy influenced overall patient-level outcomes. Univariable logistic regression identified specific sites associated with failure to achieve a CR, and these were incorporated into a multivariable model (Table 2). Adrenal/genitourinary, lungs/pleura/pericardium, and infradiaphragmatic nodes emerged as independent risk factors for not achieving a CR.

**Table 2:** Risk of key outcomes based on disease site involvement at last assessment before CAR-T therapy.

Sites^1^	Not achieving a CR^2^	PFS^3^	OS^3^
Infradiaphragmatic nodes	OR 2.13 (1.18–3.91, *p* = 0.013)	HR 1.32 (0.92–1.90, *p* = 0.13)	HR 1.44 (0.94–2.21, *p* = 0.092)
Spleen	NA^4^	NA^4^	HR 0.62 (0.27–1.42, *p* = 0.3)
CNS/cranial sinuses/orbital	NA^4^	HR 1.80 (1.06–3.08, *p* 0.031)	HR 1.85 (1.05–3.26, *p* < 0.034)
Lungs/pleura/pericardium	OR 2.34 (1.14–64.87, *p* = 0.021)	NA^4^	NA^4^
Gastrointestinal/peritoneum	OR 1.88 (0.96–3.73, *p* = 0.067)	HR 1.34 (0.91–2, *p* = 0.14)	HR 1.62 (1.03–2.55, *p* = 0.037)
Adrenal/genitourinary	OR 2.61 (1.09–6.53, *p* = 0.033)	HR 3.35 (2.16–5.19, *p* < 0.001)	HR 2.59 (1.58–4.23, *p* < 0.001)
Hepatobiliary/pancreas	OR 2.04 (0.76–5.74, *p* = 0.2)	HR 1.63 (1.01–2.63, *p* = 0.047)	HR 1.99 (1.19–3.33, *p* < 0.009)
Bone/soft tissue	NA^4^	HR 1.03 (0.71–1.50, *p* = 0.9)	NA^4^

For each site, the reference for comparison is non-involvement of the same site at the last assessment before CAR-T. The reference group for all comparisons is no involved in the specified site. 1. Sites were selected based on association with the outcome in univariable regression models. Models presented in table are multivariable adjusted for age, LBCL transformation, pre-lymphodepletion LDH, bridging, and CAR-T product. 2. Logistic regression model. 3. Cox-regression. 4. Multivariable regression models are performed if there was a significant association (p < 0.05) in univariable regression.

Using the same analytical framework, we examined PFS and OS. Adrenal/genitourinary, CNS/orbital/cranial sinuses, and hepatobiliary/pancreas involvement were associated with an increased risk of shorter PFS. Similarly, adrenal/genitourinary, hepatobiliary/pancreas, CNS/orbital/cranial sinuses, and gastrointestinal/peritoneum involvement were linked to shorter OS.

We performed a subanalysis considering CNS involvement at the last disease assessment before CAR-T, without combining it with orbital or cranial sinusoidal involvement. In multivariable Cox regression models, CNS involvement was associated with shorter PFS (HR 4.05 [95% CI: 2.14–7.67], *p* < 0.001) and OS (HR 3.60 [1.84–7.06], *p* < 0.001) compared to no CNS involvement.

### Patterns of site-specific relapse and progression following CAR-T therapy

**Fig. 4:**
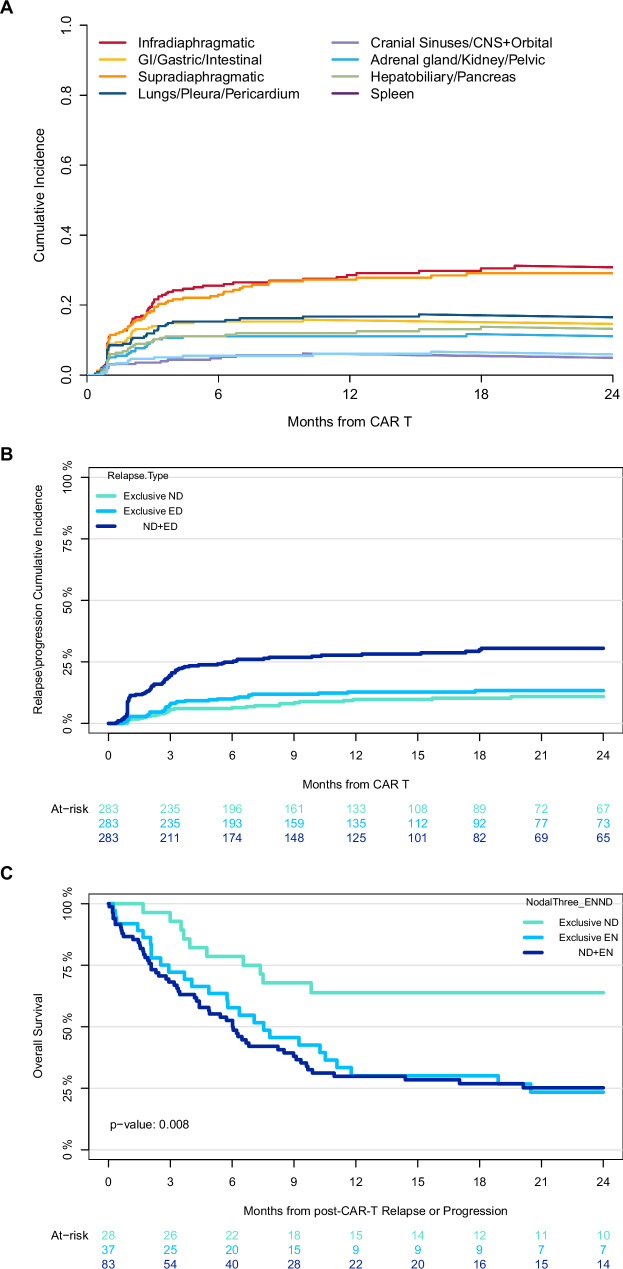
Disease involvement by site at relapse and progression after CAR-T therapy. **A** Cumulative incidence of relapse and progression after CAR-T by disease site. **B** Cumulative incidence of nodal and extranodal relapse and progression after CAR-T therapy. **C** Overall survival measured from the time of relapse or progression after CAR-T therapy.

At the time of relapse or progression, the most frequently involved sites were infradiaphragmatic nodes (53%), supradiaphragmatic nodes (50%), and bone/soft tissue (48%) (Fig. 3A). The cumulative incidence of relapse or progression at a specific site at 2 years was highest for infradiaphragmatic nodes (31%), supradiaphragmatic nodes (29%), and lungs/pleura/pericardium (17%) (Fig. 4A).

The likelihood of relapse or progression in a specific site post-CAR-T was strongly associated with prior disease involvement at that location. Lungs/pleura/pericardium (HR 7.80, [95% CI: 4.14-14.7], *p* < 0.001), gastrointestinal/peritoneum (HR 5.97, [3.07–11.6], *p* < 0.001), and bone/soft tissue (HR 5.03, [2.72–9.29], *p* < 0.001) had the highest risk of same-site involvement after CAR-T therapy compared to no baseline involvement in that site (Table 3). Comparative risk of same-site relapse or progression was not evaluable for CNS/orbital/cranial sinuses, adrenal/genitourinary, hepatobiliary/pancreas, and spleen due to an insufficient number of events. However, there was a significant association between pre-CAR-T involvement at these sites and relapse or progression at the same location (*p* < 0.001 for all).

**Table 3:** Risk of site-specific relapse or progression based on pre-CAR-T cell therapy involvement, compared to patients with no involvement at that site.

Site	Hazard Ratio (95%CI)^1^	*p*-value
Supradiaphragmatic nodes	3.04 (1.75-5.27)	<0.001
Infradiaphragmatic nodes	5.38 (2.88-10.0)	<0.001
Bone/soft tissue	5.03 (2.72-9.29)	<0.001
Gastrointestinal/peritoneum	5.97 (3.07-11.6)	<0.001
Lungs/pleura/pericardium	7.80 (4.14-14.7)	<0.001

1. Univariable cause-specific Cox regression.

### Patients with EN disease involvement after CAR-T therapy have shorter overall survival

The 1-year cumulative incidence of exclusive ND, exclusive EN, and concomitant EN and ND disease relapse or progression was 43% (95% CI: 32–54%), 48% (35–60%), and 60% (50–68%) (Fig. 4B). Importantly, in univariable Cox regression, patients with exclusive EN disease (HR 2.14, 95% CI: 1.10–4.17) or concomitant ND and EN (HR 2.52, 95% CI: 1.38–4.60, *p* = 0.003) at relapse or progression had a shorter OS from the time of relapse or progression compared to those with exclusive ND (Fig. 4C). Corresponding 1-year OS after relapse were 64% for ND (48–85%), 30% for EN (95% CI: 18–51%) and 30% for concomitant EN and ND (21%–42%).

## Discussion

In this comprehensive analysis of over 900 PET-CT scans from 283 patients with LBCL, we observed a high burden of EN disease prior to CAR-T therapy, exceeding 75%. Importantly, our findings indicate that concomitant EN and nodal disease, rather than isolated EN disease, is associated with poorer outcomes after CAR-T therapy. We also identified site-specific variations in treatment response and outcomes at both the patient and organ levels, suggesting that certain anatomical sites may act as sanctuary regions from CAR-T activity. Finally, patients experiencing relapse or progression with any EN involvement after CAR-T therapy have shorter survival than those with ND relapse. These observations underscore the need for personalized treatment strategies to address and minimize EN disease both before and after CAR-T therapy.

The current understanding of EN disease impact in CAR-T therapy remains limited, with few studies specifically examining this aspect. The largest report to date,^d5^ involving 126 patients with LBCL treated primarily with tisa-cel (79%), described reduced PFS in those with three or more EN sites before CAR-T therapy. Our analysis is considerably larger and includes patients treated across all commercially available CAR-T products, offering a more detailed understanding of EN disease’s role in CAR-T therapy. Our analysis provides several novel insights important to patient care. First, EN disease is a negative prognostic factor but primarily in the presence of concurrent nodal disease. Second, certain EN sites, such as adrenal/genitourinary, CNS, and hepatobiliary/pancreas are linked with shorter PFS and OS compared to other sites. Third, EN sites tend to persist despite bridging therapy and recur in previously involved locations. Finally, when patients relapse with EN involvement, their survival is considerably shorter than that of counterparts who relapse or progress without EN disease. Therefore, strategies prioritizing comprehensive disease debulking, particularly for EN involvement before CAR-T therapy, are warranted.

Beyar Katz et al. [25] reported a tendency for higher rates of CRS and ICANS in patients with EN disease (CRS 76% vs. 57%, *p* = 0.06), consistent with our findings of increased toxicity in this population. Grade ≥2 ICANS was more frequent in patients with EN or EN + ND disease compared to ND alone. Among patients with CNS involvement, the frequency and severity of ICANS were similar to those without CNS involvement, aligning with studies suggesting that CNS involvement does not increase the risk of severe ICANS [32–34]. However, our study may have been underpowered to detect the difference. The increased toxicity observed in EN disease highlights the potential need for prophylactic strategies. While anakinra has been explored for ICANS prevention, its role remains investigational, and further studies are warranted to define its impact on patient outcomes [35].

Data on site-specific disease dynamics remain limited. For instance, a study of 32 patients reported poorer PFS and OS associated with pre-CAR-T soft tissue infiltration but lacked detailed data on other types of involvement [17]. In our cohort, however, bone or soft tissue involvement did not correlate with worse outcomes. Similarly, a study of 24 patients with gastrointestinal involvement found no difference in outcomes compared to 106 patients without such involvement before CAR-T [36]. In contrast, our analysis identified gastrointestinal or peritoneal involvement as being associated with shorter OS. Finally, our finding of shorter PFS and OS in patients with CNS involvement before CAR-T therapy aligns with previous reports identifying this site as high risk for CAR-T treatment failure [37, 38]. CNS-directed interventions may improve outcomes in this population [39].

We identified potential anatomical sites contributing to CAR-T therapy resistance, with low response rates observed in regions such as the adrenal/genitourinary system (48%), lungs/pleura/pericardium (51%), and central nervous system (58%). Disease involvement in these areas—particularly adrenal/genitourinary, hepatobiliary/pancreas, and CNS—was associated with reduced survival. These findings highlight the importance of disease localization in predicting CAR-T outcomes and tailoring management strategies. Site-specific radiotherapy may help reduce tumor burden in EN lesions before CAR-T therapy, addressing the limitations of traditional chemotherapy in accessing these sanctuary sites [6, 39–41].

EN disease burden in newly diagnosed LBCL is approximately 30% [42, 43]. However, its prevalence exceeds 75% in our cohort before CAR-T therapy, reflecting a more aggressive disease profile in these patients [15, 16], which may contribute to their enrichment in second-line or later treatments. Unique genomic and molecular features drive the tropism of LBCL to EN sites, with recurrent mutations in genes like *MYD88*, *CD79B*, and *CDKN2A* commonly described in CNS, skin, breast, and ear-nose-throat sites [20, 21, 44–46]. These shared mutations suggest molecular similarities among EN lymphomas across various locations. Molecular findings in our cohort align with previous studies, demonstrating a higher frequency of *TP53* and *KMT2D* mutations in EN disease [47, 48]. Given that biopsies typically target a single site, the full extent of intra-patient heterogeneity in EN LBCL may also be underappreciated.

This study has notable limitations, including its retrospective and single-center design. The extent of EN involvement may be underestimated, as brain MRIs were conducted in only 67% of patients, and bone marrow assessments were performed too infrequently to be included in the analysis. However, PET-CT, the standard imaging modality for LBCL evaluation, was available for all patients, ensuring a consistent assessment of disease burden. Additionally, not all patients underwent biopsies to confirm relapse, which may affect the precision of relapse data. In addition, the association between specific EN sites and poorer survival or relapse risk, while significant, could be influenced by unmeasured factors such as treatment history and prior therapies. Future studies with multi-site sampling and longitudinal designs could enhance our understanding of EN disease dynamics in LBCL and provide a more comprehensive view of disease heterogeneity.

In conclusion, this study demonstrates the significant impact of disease localization on CAR-T therapy outcomes in lymphoma, with high rates of treatment failure in cases involving adrenal/genitourinary, hepatobiliary/pancreas, and CNS sites. The adverse prognosis associated with EN disease, along with the increased incidence and severity of treatment-related toxicities, calls for potentially personalized treatment strategies and vigilant monitoring in this vulnerable group. Future research should aim to elucidate the biological mechanisms driving these disparities and develop innovative approaches to enhance outcomes for patients with EN disease.

## Supplementary information

SUPPLEMENTAL MATERIAL

## Data Availability

The datasets supporting the findings of this study, including imaging and sequencing data, are available upon reasonable request from the corresponding author, subject to institutional and ethical approvals.
