# Supplementary material for: CAR T-cell therapy response varies by extranodal disease site in large B-cell lymphoma
Source: Blood Cancer J. 2025 Apr 14;15(1):64. doi: 10.1038/s41408-025-01273-1 (PMC11997148; doi:10.1038/s41408-025-01273-1)
Supplement: Supplementary file 1 — SUPPLEMENTAL MATERIAL [file 41408_2025_1273_MOESM1_ESM.docx]

**Figure S1. Alluvial plot of nodal and extra-nodal disease involvement before and after apheresis in patients receiving systemic bridging therapy.**


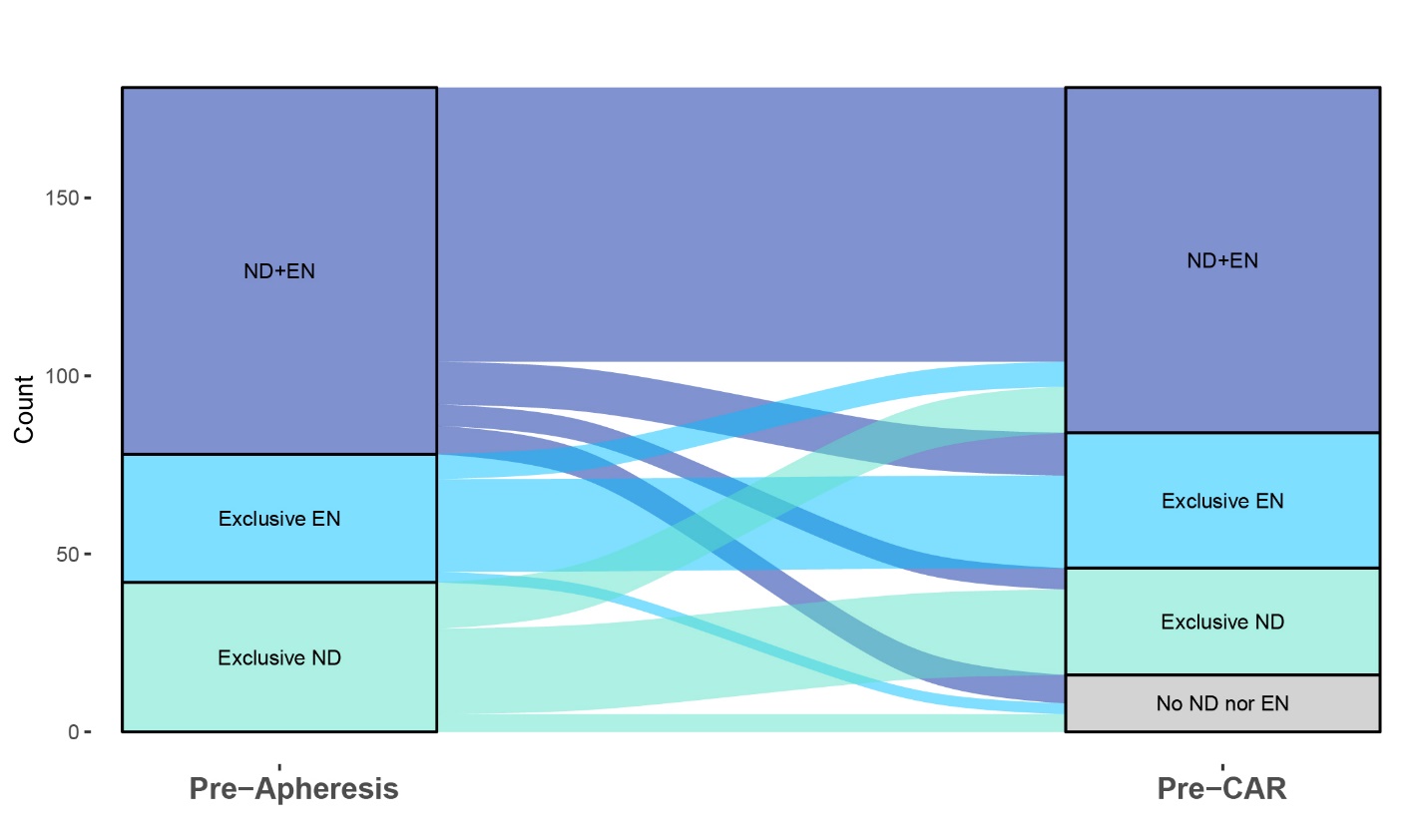


Nodal disease (ND); Extranodal disease (EN), concomitant nodal and extranodal disease (ND+EN).

**Figure S2. Genomic landscape of LBCL by extranodal disease involvement before apheresis.**


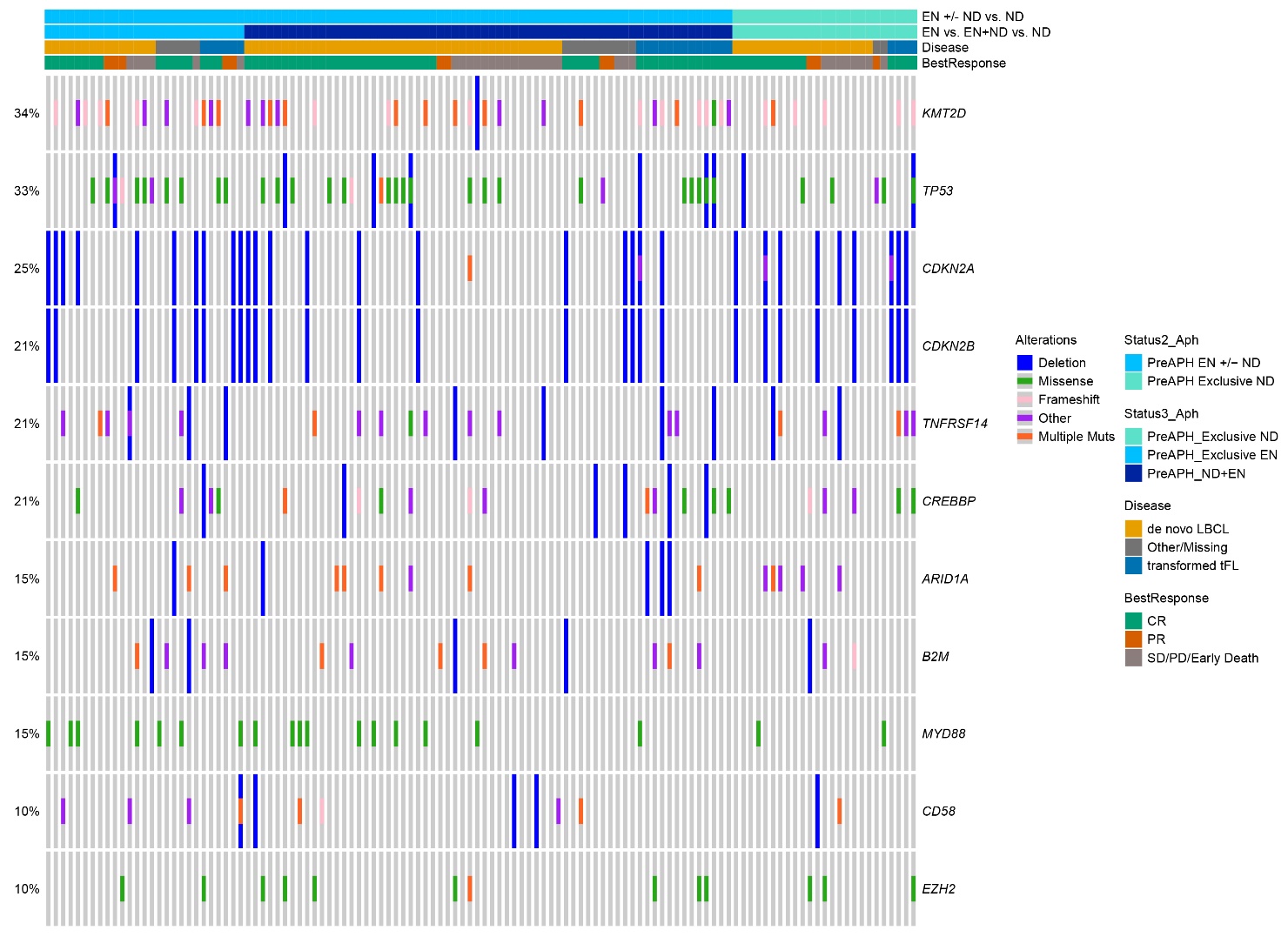


LBCL: Large B-cell Lymphoma, tFL: transformed follicular lymphoma. CR: complete response. PR: partial response. SD: stable disease. PD: progressive disease.

**Table S1. Commonly altered genes by EN status before apheresis.**

| Gene altered | Overall patients evaluable (n=118) | Any EN disease (n=93) | Exclusive ND disease (n=25) | p-value^2^ | q-value^3^ |
| --- | --- | --- | --- | --- | --- |
| *KMT2D* | 43 (36%) | 37 (40%) | 6 (24%) | 0.15 | 0.6 |
| *TP53* | 41 (35%) | 35 (38%) | 6 (24%) | 0.2 | 0.6 |
| *CDKN2A* | 31 (26%) | 22 (24%) | 9 (36%) | 0.2 | 0.6 |
| *CDKN2B* | 27 (23%) | 19 (20%) | 8 (32%) | 0.2 | 0.6 |
| *TNFRSF14* | 27 (23%) | 20 (22%) | 7 (28%) | 0.5 | 0.9 |
| *CREBBP* | 26 (22%) | 21 (23%) | 5 (20%) | 0.8 | 0.9 |
| *ARID1A* | 19 (16%) | 14 (15%) | 5 (20%) | 0.5 | 0.9 |
| *B2M* | 19 (16%) | 16 (17%) | 3 (12%) | 0.8 | 0.9 |
| *MYD88* | 19 (16%) | 17 (18%) | 2 (8.0%) | 0.4 | 0.8 |
| *CD58* | 13 (11%) | 11 (12%) | 2 (8.0%) | 0.7 | 0.9 |
| *EZH2* | 13 (11%) | 10 (11%) | 3 (12%) | >0.9 | >0.9 |

1. Pearson's chi-squared test; Fisher's exact test; 2. False discovery rate correction for multiple testing.

**Table S2. Outcomes at two years after infusion by number of extranodal lesions determined at last assessment before CAR-T infusion.**

| Outcome | 1 EN site | 2 EN sites | 3 or more EN sites | p-value |
| --- | --- | --- | --- | --- |
| PFS-2 years | 53% (41%, 70%) | 49% )31%,75%) | 26% (17%, 41%) | 0.003^1^ |
| OS-2 years | 55% (42%, 71%) | 56% (40%, 79%) | 34% (24%, 47%) | 0.016^1^ |
| Relapse-2 years | 53% (39%, 65%) | 67% (41%, 83%) | 75% (63%, 83%) | 0.024^2^ |

1. Log-rank test; 2. Gray’s test

**Table S3. Association between the number of extranodal disease sites before CAR-T therapy and treatment outcome in multivariable regression models^1^.**

| Number of Extranodal sites | Not achieving a CR | PFS | OS | Relapse |
| --- | --- | --- | --- | --- |
| 1 site | Reference | Reference | Reference | Reference |
| 2 site | OR 0.70 (0.27-1.76, p=0.5) | HR 1.38 (0.80-2.38 , p=0.2) | HR 1.10 (0.56-2.18, p=0.8) | HR 1.36 (0.77-2.42, p=0.3) |
| 3 or more sites | OR 2.39 (1.18-4.96, p=0.017) | HR 1.66 (1.07-2.57, p=0.023). | HR 1.77 (1.08-2.89, p=0.024) | HR 1.76 (1.12-2.76, p=0.015_ |

1. Multivariable regression models (logistic regression for CR and Cox regression for the remaining outcomes) were adjusted for age, transformed lymphoma, pre-lymphodepletion LDH, bridging, and CAR-T product.

**Table S4. Bridging therapies by extranodal status.**

| Type of bridging | Exclusive ND | Exclusive EN | ND+EN | No evidence of disease (CR) |
| --- | --- | --- | --- | --- |
| Polatuzumab-based | 17 (27%) | 18 (27%) | 35 (26%) | 7 (35%) |
| Intensive chemotherapy | 7 (11%) | 10 (15%) | 33 (25%) | 7 (35%) |
| Radiotherapy | 17 (27%) | 14 (21%) | 15 (11%) | 2 (10%) |
| Steroids monotherapy | 1 (1.6%) | 1 (1.5%) | 8 (6.0%) | 0 (0%) |
| Other categories | 5 (7.8%) | 12(18.2%) | 24 (18%) | 2 (10%) |
